# Antioxidant-containing monoolein aqueous dispersions: a preliminary study

**DOI:** 10.1007/s13346-022-01119-4

**Published:** 2022-01-27

**Authors:** Maddalena Sguizzato, Markus Drechsler, Anna Baldisserotto, Rita Cortesi, Elisabetta Esposito

**Affiliations:** 1 Department of Chemical, Pharmaceutical and Agricultural Sciences (DoCPAS), University of Ferrara, I-44121 Ferrara, Italy; 2 Bavarian Polymer Institute (BPI) Keylab “Electron and Optical Microscopy”, University of Bayreuth, 95440 Bayreuth, Germany; 3 Department of Life Sciences and Biotechnology (SVEB), University of Ferrara, I-44121 Ferrara, Italy

**Keywords:** Monoolein aqueous dispersions, Cubosomes, Vesicles, Vitamin E, Vitamin C, Nanoparticles

## Abstract

The present study describes a preliminary study on the use of monoolein aqueous dispersions (MADs) as delivery systems for antioxidant molecules, namely, ascorbyl palmitate (AP) and alpha-tocopherol (AT). MAD, produced by emulsifying monoolein (4.5% w/w) in water and poloxamer 407 (0.5% w/w) as emulsifier, was characterized in terms of size, morphology, and antioxidant activity by mean of PCS, cryo-TEM, and (2,2-diphenyl-1-picrylhydrazyl) assay. MAD-AP or MAD-AT gave rise to a bimodal size distribution with mean size around 200 nm. All the preparations stored at 25 °C showed quite stable size at least up to 90 days. Cryo-TEM images confirmed MAD size distribution and indicated different MAD morphologies as a function of the loaded antioxidant molecule. Indeed, in the case of MAD-AP, vesicles and cubosomes with the typical inner cubic structure were observed, while vesicles and hexosomes were shown for MAD-AT. The encapsulation efficiency of both antioxidants reached more than 90% with respect to the total amount of drug used for MAD preparation. Moreover, AP and AT antioxidant activity was retained after encapsulation, and in vitro Franz cell experiments showed that the MAD enabled to better control the drug release. These preliminary results suggest that MAD formulations could be further investigated as a potential delivery system for antioxidant supplementation in dietary or cosmetic fields.

**Graphical abstract:**

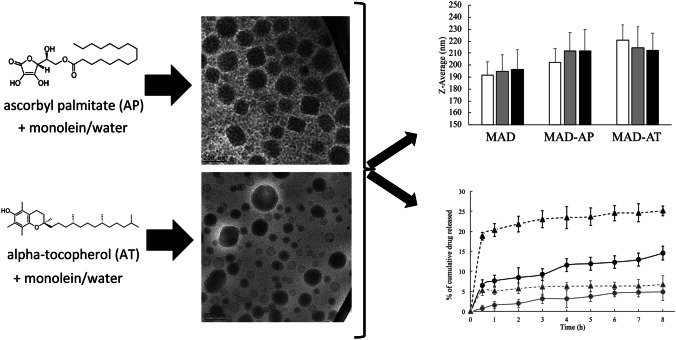

## Introduction

The amphiphilic substance glyceryl monoolein when poured in aqueous media can self-associate to form monoolein aqueous dispersions (MADs) able to provide for sustained drug release. These obtained heterogeneous systems are characterized by a mixture of complex lyotropic liquid crystalline nanostructures (LLCNs) such as micelles, cubic, lamellar, reverse hexagonal, and hexagonal mesophases [1, 2].

Indeed, unsaturated long-chain monoglycerides dispersed in water allow the formation of physically stable and biocompatible bicontinuous cubic phases [3] that can be proposed for various routes of administration such as oral, parenteral, and percutaneous leading to possible control of the release of drugs [4]. In addition, due to their structural characteristics, LLCN can allow the partition of amphiphilic, hydrophobic, and hydrophilic molecules, both concurrently or separately as a function of molecule polarity [5, 6]. Indeed in literature, many examples of solubilizing lipophilic additives, such as tocopherol, tocopherol acetate, and vitamin K, into LLCN are reported [6–9].

In the present study, we investigated the production, characterization, and stability of monoolein aqueous dispersions (MADs) as delivery systems for antioxidant molecules, such as ascorbyl palmitate (AP) and alpha-tocopherol (AT), for the potential topical administration on skin or mucosae.

AP, as hydrophobic derivative of vitamin C, was investigated in reason of its potential antioxidant activity used in many fields such as biochemistry, medicine, and food. Indeed, AP is an efficient reducing agent able to protect organic and biological molecules against oxidative degradation [10]. On the other hand, AT is a natural lipophilic compound inhibiting the peroxidation of membrane lipids, therefore protecting the cells from damaging effects [11]. Moreover, it is also known to stabilize biological membranes by mean of van der Waals interactions with phospholipids [8].

The strategy of incorporating these two model antioxidant drugs in MAD formulations could be an interesting approach in order to obtain a formulation suitable for antioxidant supplementation in food and mainly in cosmetics.

## Materials and methods

### Materials

Glyceryl monooleate RYLO MG 19 (monoolein) was a gift from Danisco Cultor (Grindsted, Denmark). Pluronic F127 (Poloxamer 407, poloxamer) (PEO_98_-POP_67_-PEO_98_) was obtained from BASF (Ludwigshafen, Germany). DPPH (2,2 diphenyl-picryl-hydrazyl), ascorbyl palmitate (AP), alpha-tocopherol (AT), and HPLC solvents were purchased from Sigma-Aldrich, Merck (Milano, Italy).

### MAD preparation

Preparation of MAD water dispersions was based on the monoolein and poloxamer emulsification process as previously described [12, 13]. Briefly, after dispersing monoolein and poloxamer 407 in water at ratios reported in Table 1, the dispersion was homogenized at 60 °C and 15,000 rpm/min-1 (Ultra Turrax, Janke & Kunkel, Ika-Werk, Sardo, Italy) for 5 min, then cooled and maintained at room temperature in glass vials.

**Table 1:** Composition and encapsulation efficiency of the produced unloaded and loaded MAD

**Formulation**	**Composition % (w/w)**					**Encapsulation efficiency (% weighted amount)**
	**Glyceryl monooleate**	**Poloxamer 407**	**Water**	**AP**	**AT**	
MAD	4.5	0.5	95	–	–	–
MAD-AP	4.5	0.5	95	0.2	–	91.1 ± 1.23
MAD-AT	4.5	0.5	95	–	0.2	96.4 ± 0.98

To produce AP and AT containing MAD, 2 mg/ml of AP and AT corresponding to 0.2% by weight of the total dispersion was employed. AP and AT were added to the molten monoolein/emulsifier mixture and dissolved before addition to the aqueous solution. Afterward, the dispersion was prepared as above described.

### Characterization of MAD

#### PCS

Submicron particle size analysis was performed using a Zetasizer Nano Series, Nano SP90 (Malvern Instr., Malvern, England) equipped with a 5 mW helium neon laser with a wavelength output of 633 nm. Glassware was cleaned of dust by washing with detergent and rinsing twice with water for injections. Measurements were made at 25 °C at an angle of 90°. Data were interpreted using the CONTIN method [14].

#### Cryo-transmission electron microscopy (Cryo-TEM)

Samples vitrified as previously described [15] were transferred to a Zeiss EM922Omega transmission electron microscope for imaging using a cryoholder (CT3500, Gatan). Throughout the examination, the sample temperature was kept below −175 °C. Specimens were examined with doses of about 1000–2000 e/nm^2^ at 200 kV. Images recorded by a CCD digital camera (Ultrascan 1000, Gatan, Munich, Germany) were analyzed by mean of GMS 1.8 software (Gatan, Munich, Germany).

### Drug content in MAD

The encapsulation efficiency (EE) of AP and AT in MAD was determined as previously described [16]. One hundred microliters of each MAD batch was loaded in a centrifugal filter (Microcon centrifugal filter unit YM-10 membrane, NMWCO 10 kDa, Sigma Aldrich, St Louis, MO, USA) and centrifuged (SpectrafugeTM 24D Digital Microcentrifuge, Woodbridge NJ, USA) at 8000 rpm for 20 min.

The amount of entrapped AP and AT was determined by dissolving the lipid phase in the supernatant with a known amount of methanol (1:10, v/v), while the amount of free drug was determined in the filtrated aqueous phase. The samples were then filtered through 0.45 μm membrane pore size and analyzed by HPLC as detailed below. All data were the mean of 3 determinations on different batches of the same type of dispersion. EE was determined applying Eq. (1):

1 $$\mathrm{EE}\:=\:\mathrm{amount}\;\mathrm{of}\;\mathrm{AP}\;\mathrm{or}\;\mathrm{AT}\;\mathrm{detected}\;\mathrm{in}\;\mathrm{the}\;\mathrm{lipid}\;\mathrm{phase}\times100/\mathrm{total}\;\mathrm{amount}\;\mathrm{of}\;\mathrm{AP}\;\mathrm{or}\;\mathrm{AT}\;\mathrm{weighted}$$

HPLC analyses were performed using a HPLC system Series 1200 (Agilent Technologies Italia, Milan, Italy) equipped with a two-plungers alternative pump (Jasco Corporation, Cremella, Italy) and a UV-detector at 254 nm and 295 nm respectively for AP and AT. Then, 50 μL samples were injected by means of a 7125 Rheodyne injection valve with a 50-μL loop on a stainless steel Kinetex^®^ C18 reverse-phase column (150 mm × 4.6 mm) packed with 5-μm particles (Phenomenex Srl, Milan, Italy). Injections were repeated thrice. Elution of drugs was performed with a mobile phase containing methanol (85%) and water (15%) flowing at a rate of 1 mL/min.

### In vitro release profiles studies

In vitro release profiles studies were performed using Franz cells (Vetrotecnica, Padova, Italy) associated to nylon membrane (Millipore, 0.45 μm pore size).

Nylon membranes were hydrated by immersion in distilled water at room temperature for 1 h before being mounted on Franz cell. The exposed surface area was 0.78 cm^2^ (the diameter of the orifice was 1 cm). The receptor compartment contained 5 ml of a mixture of phosphate buffer 60 mM pH 7.4 and ethanol (50:50, v/v). This solution was stirred with the help of a magnetic bar at 500 rpm and maintained at 32 ± 1 °C during all the experiments [17].

One milliliter of each formulation was placed on the membrane surface then the donor compartment was sealed to avoid evaporation. At predetermined time intervals comprised between 1 and 8 h, namely, 30 min, 1 h, 2 h, 3 h, 4 h, 5 h, 6 h, 7 h and 8 h, 0.250 ml samples of receptor phase were withdrawn and subjected to HPLC analyses to measure AP or AT concentration. Each withdrawn sample was replaced with an equal volume of fresh receptor phase. The drug concentration was determined three times in independent experiments, and the mean values ± standard deviations were calculated. The mean values were then plotted as a function of time. As reference, AP and AT (2 mg/ml) solutions in phosphate buffer 60 mM pH 7.4 and ethanol (50:50, v/v) were used.

### Stability studies

Physical and chemical stability studies were conducted in triplicate during 90 days from formulations production.

Physical stability was evaluated by visual inspection of the macroscopic aspect (i.e., phase separation, turbidity, and macroscopic viscosity) of the formulations, and by PCS. In particular, dispersion size was evaluated up to 40 days on MAD stored at 4 °C, 25 °C, and 37 °C; whereas chemical stability was evaluated on drug-loaded formulations, stored at 25 °C, determining AP and AT content by HPLC analyses.

### 2-diphenyl-1-picrylhydrazyl assay

The ability of the AP and AT to scavenge 2,2-diphenyl-1-picrylhydrazyl (DPPH) free radical was assessed by the standard assay able to detect compounds acting for transfer of hydrogen or electrons (radical quenching) [18, 19]. Such ability is evaluated by measuring the decrease of absorbance at 517 nm of DPPH (red–purple colored) after the radical reaction with the products to be tested. Indeed, DPPH solution decolorizes when an antioxidant agent is added. The percentage of radical scavenging capacity was calculated using Eq. (2):

2 $$\mathrm{DPPH}\;\mathrm{radical}-\mathrm{scavenging}\;\mathrm{capacity}\;(\%)=\lbrack1-(\mathrm A1-\mathrm A2)/\mathrm A0\rbrack\times100$$

where *A*0 is the absorbance of the control (without AP or AT), *A*1 is the absorbance in the presence of the AP or AT, and *A*2 is the absorbance without DPPH.

To 1.5 mL of DPPH methanol solution, 0.750 mL of AP or AT (alone or MAD) at different concentration was added. The absorbance at 517 nm was measured by a UV–VIS spectrophotometer (UV-31 SCAN ONDA, Sinergica, Milano, Italy) according to a described procedure [20]. AP and AT methanol solutions (namely Sol-AP and Sol-AT) were used as comparison. The IC_50_ values, obtained from at least three different experiments, were expressed as µg/mL (mean value ± the standard deviation) and determined by regression analysis of the results obtained at different sample concentrations.

### Statistical analysis

Statistical analysis was performed by the analysis of variance (ANOVA). The level of significance was taken at *p*-values < 0.05.

## Results and discussion

### Production of MAD

In the present paper, MADs have been investigated as suitable vehicle for the delivery of AP and AT via topical application, and a preliminary study concerning the production, the characterization, and the stability of the formulations has been conducted. As widely described, the encapsulation of these molecules represents a challenge in the increase of solubility, being highly lipophilic, as well as in preserving their antioxidant potential, highly sensitive to oxidation and external factors, such as air and light [1, 12, 13, 21, 22].

MADs have been prepared following the emulsification method. Briefly, monoolein has been dispersed in a water solution of poloxamer 407 used as emulsifier. Following the composition described in Table 1, the antioxidant molecule AP or AT has been dispersed in the lipid phase. With respect to the total amount of weighted drug, the encapsulation efficiency values were found to be 91% and 96% for AP and AT, respectively (Table 1).

In the field of nanotechnology, MADs have been recently widely investigated due to the intrinsic potential of monoolein dispersed in water, resulting in heterogeneous populations of nanostructures able to solubilize high amounts of drugs [3, 23]. The presence of poloxamer 407 in the composition, due to its surfactant properties, allowed obtaining physically stable formulation with no aggregation phenomena and phase separation. In addition, the presence of the drug at the concentration of 0.2% w/w within the loaded-formulations MAD-AP or MAD-AT did not affect the macroscopic aspect of the dispersions suggesting an effective composition.

### Characterization of MAD

#### Size distribution of MAD

**Fig. 1:**
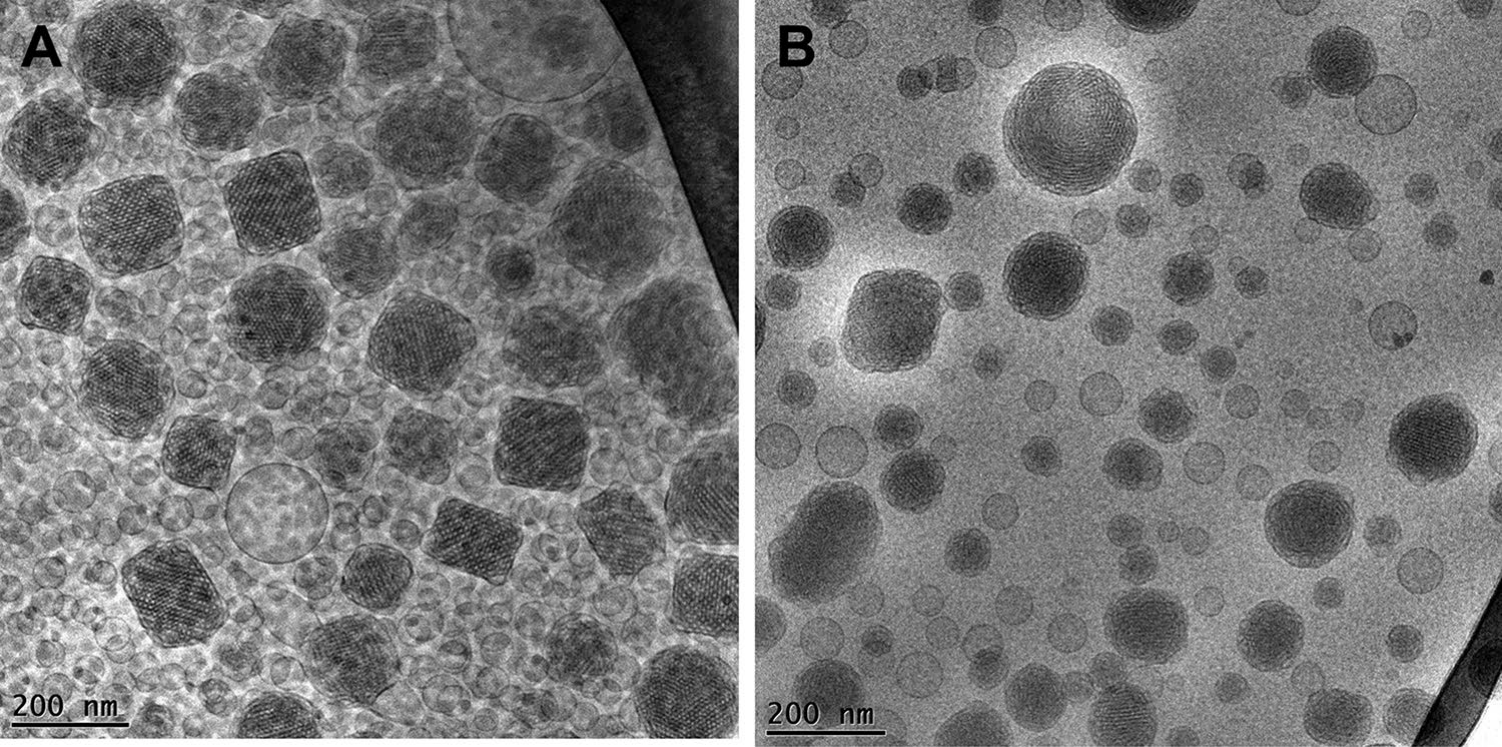
Representative Cryo-transmission electron microscopy (Cryo-TEM) images of MAD-AP (**A**) and MAD-AT (**B**)

Unloaded and loaded MADs have been analyzed by PCS in order to get information about the dimensions and the distribution of the dispersed nanosystems. Table 2 summarizes the size and the polydispersity of MAD as *Z*-average (nm) and PDI, respectively. As reported, all the formulations after production are characterized by sizes around 200 nm with a slight increase of the mean diameter in the case of loaded-MAD, being 192 nm, 202 nm, and 221 nm for MAD, MAD-AP, and MAD-AT, respectively. Hence, the presence of the encapsulated molecules probably led to a rearrangement of the lipid crystalline state generating greater structures, as evidenced by the presence of hexosomes (in some cases larger than cubosomes) from the cryo-TEM images (Fig. 1).

**Table 2:** Dimensional parameters expressed as *Z*-average (nm) and polydispersity index (PDI) of MAD, MAD-AP, and MAD-AT in time

**Day**	**MAD**	**MAD-AP**	**MAD-AT**
	***Z*-average (nm)**	***Z*-average (nm)**	***Z*-average (nm)**
	***PDI***	***PDI***	***PDI***
0	191.8 ± 10.93	202.2 ± 11.53	220.8 ± 12.59
	*0.21* ± *0.01*	*0.19* ± *0.02*	*0.22* ± *0.02*
3	195.4 ± 14.97	212.2 ± 14.22	220.4 ± 14.94
	*0.29* ± *0.05*	*0.23* ± *0.02*	*0.25* ± *0.04*
7	192.9 ± 11.26	212.7 ± 10.42	222.7 ± 18.08
	*0.28* ± *0.03*	*0.27* ± *0.01*	*0.26* ± *0.07*
15	197.4 ± 15.61	214.8 ± 12.63	221.9 ± 21.99
	*0.23* ± *0.01*	*0.26* ± *0.04*	*0.22* ± *0.06*
21	199.2 ± 16.93	209.3 ± 18.84	213.9 ± 11.12
	*0.27* ± *0.07*	*0.33* ± *0.06*	*0.28* ± *0.01*
30	206.6 ± 14.01	211.8 ± 15.25	214.4 ± 17.58
	*0.32* ± *0.01*	*0.29* ± *0.03*	*0.27* ± *0.05*
40	191.1 ± 13.94	211.1 ± 11.61	212.2 ± 11.03
	*0.25* ± *0.03*	*0.26* ± *0.04*	*0.31* ± *0.02*

Concerning the size distribution, no differences have been detected in the case of unloaded or loaded formulations. The low values of PDI, below 0.3, indicated a bimodal distribution with homogeneous formulations, as observed from a macroscopic point of view [24].

Furthermore, size and polydispersity of formulations stored at room temperature indicated stable dimensional performances. Notably, the use of poloxamer 407 as emulsifier sterically stabilizes monoolein and assures its interaction with the encapsulated drugs [25].

### Morphological characterization by Cryo-TEM

In order to shed light on the effect of the composition and the encapsulated antioxidant on the morphology of the dispersions, Cryo-TEM analysis has been conducted. The loaded-formulations have been visualized, and the obtained images of MAD-AP (panel A) and MAD-AT (panel B) are reported in Fig. 1.

In both images, a heterogeneous nano-sized population composed of uni- and multilamellar vesicles, cubosomes, and hexosomes could be evidenced. Particularly, vesicles and cubosomes with the typical cubic inner configuration represent the more abundant structures of the formulation MAD-AP, while in MAD-AT, unilamellar vesicles and hexosomes are mainly detected. Taking into consideration the chemical structure of AP and AT, the presence of miscellaneous nanostructures in the dispersions could be ascribed to the different interaction of the encapsulated drugs with monoolein and poloxamer, resulting in a mixture of crystalline phases.

### In vitro release studies

**Fig. 2:**
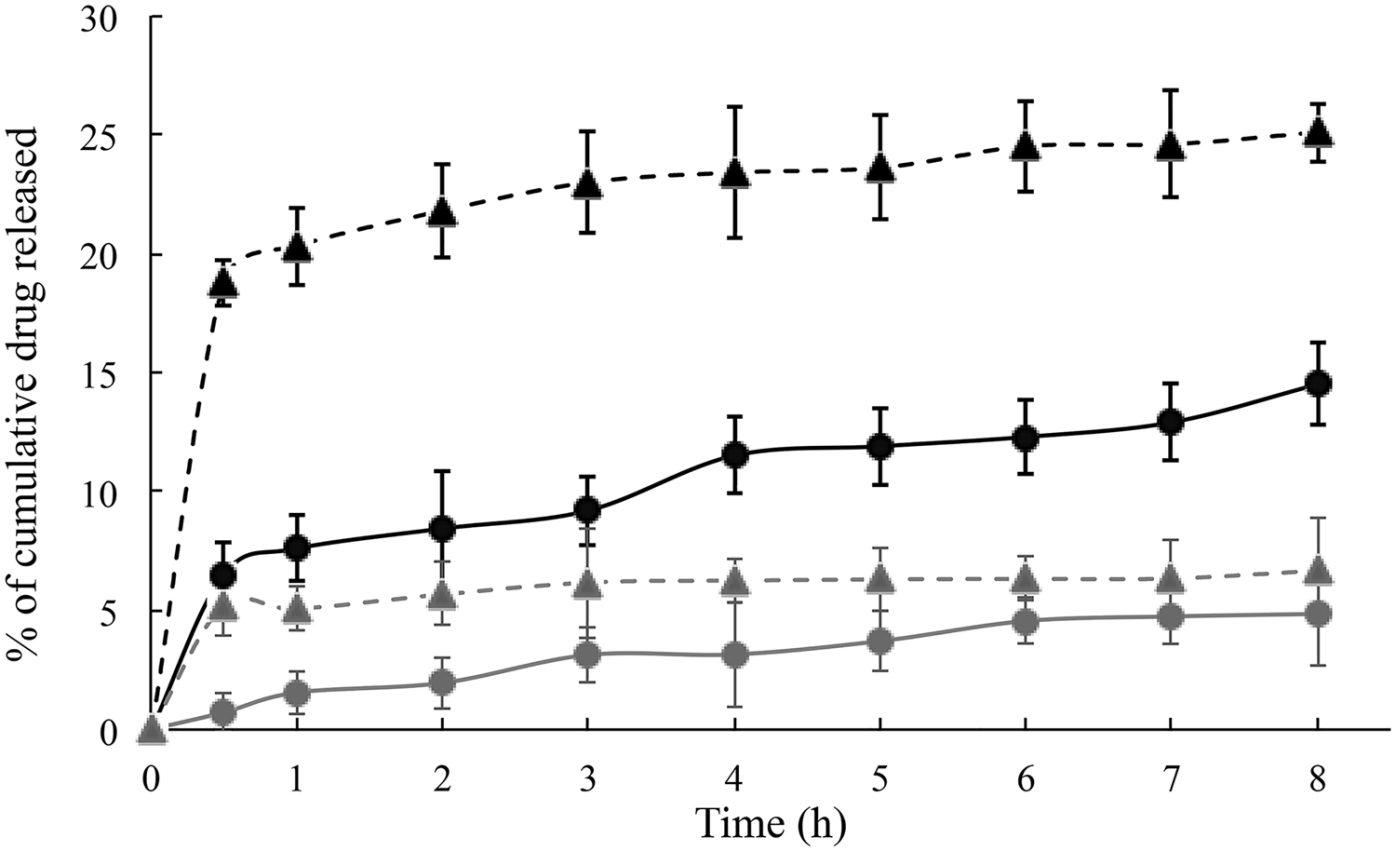
Release profiles of AP (black) and AT (grey) from MAD (circles) or solution (triangles) during time determined in vitro by using Franz cells

In vitro release experiments have been conducted using Franz cells, in order to get information about the influence of MAD morphology on drug release. It should be underlined that the lipophilic nature of AP and AT limits the diffusion of the drug in aqueous media; hence, a non-physiological receptor phase composed of ethanol and water 50:50 has been used. Particularly, a nylon membrane allowed support to the formulation without influencing the transfer of active compound to the receiving compartment, where the receptor phase guaranteed its solubilization in sink conditions [17, 26]. Figure 2 displays the release profiles of AP and AT when entrapped in MAD or as free drug solution, expressed as the percentage of cumulative drug released during time.

In both cases, MAD enabled to better control the release of the loaded drug, as compared to the corresponding drug solution, showing similar behavior for AP and AT. Comparing MAD-AP and MAD-AT profiles, the quantitative difference could be ascribed to the nature of the loaded compounds. In particular, the greater the lipophilicity, the lower the drug diffusion as confirmed by the LogP values of AP (LogP 6.3) and AT (LogP 10.7) [27, 28].

Moreover, taking into account the morphological aspect of the nanoparticles, the mostly presence of cubic structures in MAD-AP seem to better retain the drug in time as compared to the corresponding solution, suggesting the suitability of MAD as delivery systems for topical use. Overall, however, the influence on lipophilic drug diffusion of heterogeneous structures such as vesicles, cubosomes, and hexosomes still need to be clarified.

### Antioxidant activity

The radical scavenging ability of the encapsulated AP and AT has been evaluated after MAD production and compared to that of the corresponding free antioxidant molecule, in order to assess the efficiency of MAD to preserve their effect. DPPH test has been selected as a useful method widely employed for the evaluation of the antioxidant compounds [29], and it allows measure of the reducing activity of antioxidant molecules against the DPPH radical by a colorimetric reaction.

The IC_50_ values reported in Table 3, as indication of the AP and AT radical scavenging activity, suggest that the antioxidant potential of both compounds was retained even after encapsulation, confirming the suitability of MAD as promising vehicle for preserving their therapeutic activity. Furthermore, the IC_50_ values are in agreement with the encapsulation efficiency values reported in Table 1 and also with the in vitro release kinetics shown.

**Table 3:** Antioxidant activity of AP and AT either in solution or in MAD evaluated by DPPH analysis and expressed as IC_50_

**Formulation**	**IC**_**50**_ **(µg/ml)**
Sol-AP	18.36 ± 1.44
MAD-AP	23.30 ± 1.00
Sol-AT	21.34 ± 0.27
MAD-AT	26.04 ± 0.40

Indeed, similarly to what was previously observed, the slight decrease in the antioxidant activity of the trapped active ingredients in the MAD could be due precisely to the drug release control effect operated by the nanosystem itself.

These data corroborate the results of encapsulation efficiency in MAD above discussed. Indeed, as the total AP and AT used has been loaded in the formulations, the effect of drugs in reducing DPPH free radical is comparable to that of solutions, where they are completely solubilized. Hence, drug encapsulation in MAD allowed to make AP and AT available in a water-based matrix maintaining their chemical and biological properties.

### Stability studies

#### Physical stability

The physical stability of MAD, MAD-AP, and MAD-AT has been evaluated by submitting the formulation to visual inspection and PCS analysis. From a macroscopic point of view, all the formulations appeared white-colored and homogeneous, with no aggregates, changes in color or instability phenomena for about 3 months.

**Fig. 3:**
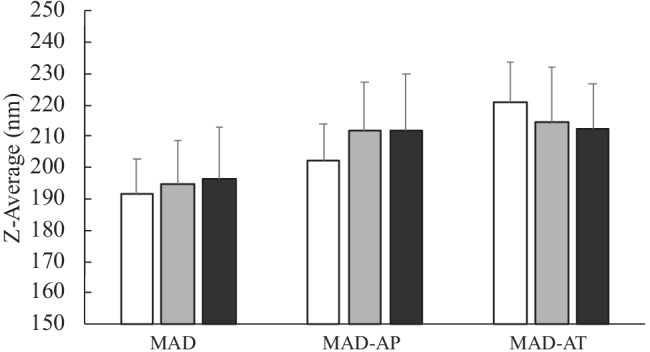
Mean size of MAD, MAD-AP, and MAD-AT, expressed as *Z*-average (nm), measures at day 1 (white), 30 (grey), and 90 (black) after production

Considering the stable dimensional distribution revealed at 25 °C, the vesicles size has been measured up to 90 days after production, and the results are shown in Fig. 3.

The mean sizes of MAD, MAD-AP, and MAD-AT have been compared 1 day, 30 days, and 90 days after production, and the results indicated stable dimensions during time. Particularly, for MAD, the size was maintained in the range of 190–200 nm, without a significant variation, whereas loaded MAD displayed a slight dimensional variation probably due to the presence of the antioxidant drug.

#### Antioxidant content

The poor solubility of AP and AT along with their sensitivity to oxidization is the most important drawbacks to overcome in order to obtain effective formulations with higher therapeutic effect. Hence, after MAD production, the antioxidant content, expressed as percentage of the total amount used for the preparation, has been monitored up to 40 days. The stability content was investigated by HPLC, and it was found that during time, both MAD-AP and MAD-AT are not able to maintain unchanged the antioxidant content, even if the encapsulation efficiency after production was almost quantitative (Table 1). Indeed, the antioxidant content is almost halved after 40 days from production (data not shown). This result suggests the need to investigate more in depth the composition and interaction of the antioxidant molecule with the lipid matrix to avoid its possible degradation or oxidation. Given the challenge to the solubility of AP and AT, MAD could indeed be considered a promising delivery system for increasing the bioavailability of these antioxidant molecules and their subsequent therapeutic activity or dietary supplementation. Nevertheless, the possibility of solubilizing AP and AT in an aqueous system is an important goal in view of a topical dermatological administration, avoiding skin irritation and toxicity.

#### Influence of storage temperature on MAD size

In MAD production, key parameters are represented by nature of lipids and emulsifiers, water content, and also temperature [23].

**Fig. 4:**
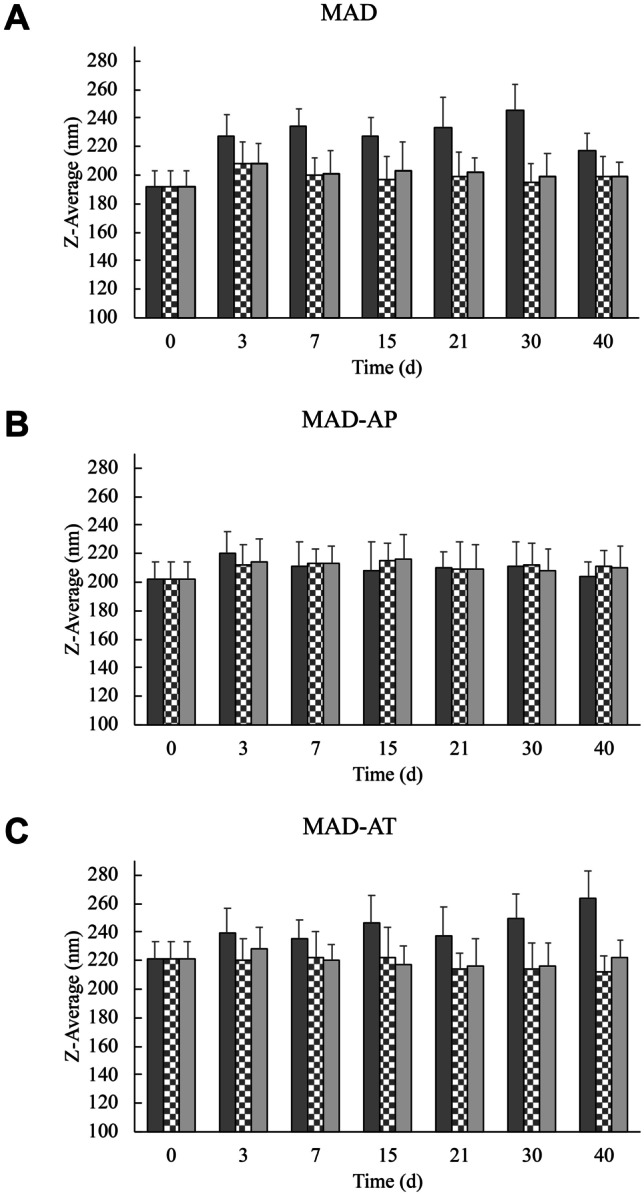
MAD (**A**), MAD-AP (**B**), and MAD-AT (**C**) dimensions expressed as *Z*-average (nm) during time. Samples were stored at 4 °C (dark grey), 25 °C (checkerboard), and 37 °C (light grey). *p < 0.05; **p < 0.01 vs corresponding MAD at time 0 and the same storage conditions

At this respect, in order to evaluate the influence of temperature on dimensional stability, MAD, MAD-AP, and MAD-AT have been stored at different conditions, namely, 4 °C, 25 °C, and 37 °C, and PCS analysis has been periodically conducted. Figure 4 graphically shows the behavior during time of MAD dimensions stored at different temperatures.

By comparing the different formulations, it emerged that MAD-APs were not affected by the storage conditions. Indeed, dimensions were stable in the range of 200–220 nm during time and different temperatures.

In the case of MAD and MAD-AT, the storage at 4 °C influenced the size stability leading to an increase in nanoparticle mean diameter, while at 25 °C and 37 °C, no significant differences were detected. This result corroborates the suitability of MAD as topical delivery systems. Indeed, on one hand, the stability of formulations at room temperature facilitates the storage conditions of MAD; on the other hand, the stability at 37 °C assures the preservation of their dimensional performance even in contact with the body temperature after topical application. Conversely, the low temperature produced lipid crystallization and conferred more rigidity to the nanostructure resulting in greater sizes. Particularly, in the case of MAD-AT, the variation of size in relation to storage condition could influence AT stability, encapsulation, and also therapeutic effect; therefore, the room temperature could be considered the optimal storage condition.

## Conclusions

This study indicates that MAD can be used as a tool to transport AP and AT in order to overcome their poor aqueous solubility. Particularly, MAD-AP and MAD-AT showed a bimodal size distribution with mean size around 200 nm that remains quite stable over 90 days from production. The type of loaded antioxidant molecule influences MAD morphologies giving rise to vesicles and cubosomes for AP and to vesicles and hexosomes for AT. However, the composition of these formulations needs to be further investigated to maintain high antioxidant content during time. On the other hand, in vitro Franz cell experiments showed that the MAD enabled to better control the release of AP and AT, as compared to their respective drug solution. In addition, the antioxidant potential was retained after encapsulation confirming the suitability of MAD as promising vehicle for preserving their therapeutic activity. Therefore, the preliminary results here described suggest that MAD formulations could be further investigated as a potential delivery system for antioxidant possibly for dietary supplementation or dermatological application.

## Data Availability

The authors confirm that the data supporting the finding of this study are available within the article. Raw data are available from the corresponding author (RC) upon reasonable request.

## Declarations

### Disclaimer

The funders had no role in the design of the study; in the collection, analyses, or interpretation of data; in the writing of the manuscript, or in the decision to publish the results.
